# The reduction of tuberculosis risks by smoking cessation

**DOI:** 10.1186/1471-2334-10-156

**Published:** 2010-06-07

**Authors:** Chi-Pang Wen, Ta-Chien Chan, Hui-Ting Chan, Min-Kuang Tsai, Ting-Yuang Cheng, Shan-Pou Tsai

**Affiliations:** 1 Division of Health Policy Research, Institute of Population Health Science, National Health Research Institutes, Zhunan, Taiwan; 2 Graduate Institute of Epidemiology, School of Public Health, National Taiwan University, Taiwan; 3 Bloomberg School of Public Health, Johns Hopkins University, Baltimore, Maryland, USA; 4 University of Texas School of Public Health, Houston, Texas, USA

## Abstract

**Background:**

Smoking is known to aggravate tuberculosis (TB), but such information has been ignored in clinical practice, as it was not thought to be relevant. The aim of this study is to assess the benefits of smoking cessation on TB mortality reduction.

**Methods:**

The study attempts to quantify smokers' risks on subsequent TB mortality and the change in such risks after smokers quit smoking. In this prospective cohort study, the TB mortality risks of smokers, never smokers and former smokers were compared, by using the Cox proportional model to estimate the hazard ratio (HR) of TB.

The cohort, consisting of 486,341 adults, participated in standard medical screening programs since 1994, including 5,036 with self-reported TB history. Of 15,268 deaths identified as of 2007, 77 were coded as TB.

**Results:**

Smokers with self-reported TB history (1.2%) had very high TB mortality (HR = 44.02). Among those without self-reported TB history, smoking increased TB mortality by nine-fold (HR = 8.56), but when they quit smoking, the risk was reduced by more than half (65%), to a level not different from those who had never smoked. The overwhelming majority of TB deaths (83%) occurred among those without self-reported TB history. Given the high smoking prevalence and the high HR, smoking accounted for more than one-third (37.7%) of TB mortality in Taiwan. Smokers reported less TB history but died more from TB than those who had never smoked.

**Conclusions:**

Smokers had very high TB mortality, as much as nine times those who had never smoked, but once they quit, the risk reduced substantially and was similar to those who never smoked. Smoking cessation has benefits to the smokers far beyond reducing TB risk, but successful tobacco control could favorably impact the TB mortality rate and reduce this public health burden, which has long haunted the Taiwanese population. Smoking cessation could reduce nearly one-third of tuberculosis deaths.

## Background

Although the relationship between smoking and TB has been extensively reviewed [1-5], most studies have determined that a strong relationship exists between smoking and TB based more on case control study design than cohort one, studying more active TB diseases than TB deaths [2]. One of the difficulties is that in countries other than India or China, deaths attributable to TB are relatively rare and the number of TB deaths is small, making statistical power difficult to reach in epidemiological studies [2]. As there was limited evidence on TB mortality from cohort studies [6-8], the clinical significance of reducing smoking on TB mortality was largely unrecognized [9]. Whether smoking was viewed as a risk factor for TB relapse was also not fully established [10]. This is due to the fact that since TB is a treatable disease[9], the role of smoking as a risk factor for TB disease was mostly perceived as irrelevant. To clinicians who focused on treating TB, reducing smoking was considered as a separate issue, not shown to be directly beneficial. The use of a cohort becomes particularly valuable because a cohort could show not only the effect of smoking in increasing the risks of TB disease [11] or mortality [12] but also it could provide the opportunity to examine TB risks among smokers who quit smoking, which would be of value for clinicians to apply in clinical practice.

Smoking caused 5 million deaths a year [13], and tuberculosis, nearly 2 million deaths a year [2]. Globally, one-third of smokers and one-fifth of TB prevalence cases (patients) are in East Asian countries in 2000 [14]. As many as one-third of the Chinese population is estimated to be infected with TB [3,15,16], known to be aggravated by smoking during its natural course [15,17]. Given that TB and smoking are two major preventable killers, it is disappointing that only recently their relationship started to receive attention in research papers, mostly from India [2,18] and East Asia [12,19,20]. The relationship took on an increasing importance in China where the interaction on outcome between tens of millions of TB patients, some known but mostly unknown, and 350 million smokers [21] is largely ignored, and therefore, the potential to reduce the large number of TB deaths in China, estimated at 200,000 in a given year, has been left unexplored [14]. Health care workers focused on TB have rarely considered the impact of smoking cessation on these patients.

The objective of this study is to assess the effect of smoking on TB mortality and the benefit of smoking cessation by following a healthy Chinese cohort. Because having TB history (or known TB patient) is a major confounder, we were particularly interested in examining the reduction of TB risks among those without TB history after they quit smoking.

## Methods

### Characteristics of the participants

The cohort, consisting of 486,341 adults, aged 20 and older, went through standard medical screening programs run by a private firm, MJ Health Management Institution, Taipei, Taiwan (MJ), since 1994. The firm attracted paying participants from all over Taiwan and across socioeconomic classes, because of its known quality and accessible key facilities. Details of the cohort have been reported elsewhere [22].

All participants had basic demographic information, and received a panel of comprehensive blood and anthropometric measurement. Body Mass Index (BMI) was calculated from weight (kg)/height (m)^2^. A written questionnaire was drafted in 1996, including one question on whether they have had TB. Questionnaire data on smoking (79%), drinking (79%) or TB history (86%) were available on a majority of the cohort subjects. The actual available data was used to arrive at the percentages.

Two levels of diabetes were identified: those by self-reported history, including taking medication for that condition, and those discovered by screening tests. The total number of diabetics was those with history and those identified by fasting glucose at or above 126 mg/dl.

### Social economic status and smoking/TB history

Education was used as surrogates for social class and classified as: middle school or lower (9 years of education or less), high school (10-12 years), junior college (13-14 years) and college or higher (16 or more years of education). Number of years and number of cigarettes smoked per day were collected from both the current smokers and ex-smokers. We used (ever smokers) to represent smokers and ex-smokers. Current smokers were those reported (smoking daily) and were asked to give number of years and number of cigarettes smoked per day. Ex-smokers were smokers who had already quit. Regular drinkers were those who drank more than two drinks each time for at least three times a week. Occasional drinkers drank less than regular drinkers. Self-reported TB history was collected from the questionnaire. Self-reported TB history was defined as being aware of or remembered of TB history when the participants were first enrolled in this study.

### Mortality rate

Taiwan has a national death file in electronic form available from the Department of Health which records deaths since 1971 [23]. With the use of unique individual ID numbers, the vital status of the cohort was obtained as of the end of 2007, with an accumulated 3,853,845 person years and a median follow-up of 8.5 years. Cause of death was coded according to 9th revision of International Classification of Disease (ICD-9). Although TB deaths included ICD-9: 010-018, all TB deaths in this study were limited to pulmonary TB (ICD-9: 011). The age-specific mortality for TB for men and women in Taiwan, for the period 1994-2007, came from the Taiwan Department of Health [23].

### Statistical analysis

The study attempts to quantify the smoker's risks on subsequent TB mortality and the change in such risks after smokers quit smoking. In this prospective cohort study, the TB mortality risks of smokers, never smokers and former smokers were compared by using the Cox proportional model to estimate the hazard ratio (HR) of TB. HRs were presented first with age and gender adjustment and then with the additional education, drinking, BMI and diabetes mellitus history adjustment. Smoking attributable fraction for TB mortality was calculated from the formula: Prevalence × (HR - 1)/{1 + [Prevalence × (HR - 1)]} where prevalence was the national prevalence for smoking in Taiwan [24].

### Ethical issues

Every participant had signed a consent form authorizing MJ to process the data. The Institutional Review Board of the National Health Research Institute processed and approved the study. Individual names were removed early in the process and the entire analyses were conducted with anonymous data.

## Results

### Self-reported TB

Characteristics of the subjects in the cohort, 486,341, were compared with those reporting positive TB history, 5,036 (1.20%), and the differences expressed as odds ratio (OR) in Table 1. Self-reported TB history increased sharply with increasing age. Men reported more TB than women by 79%. Below age 40, lower educated reported more TB history, but above age 40, higher educated reported more TB history. Because of this cross over, the relationship between TB history and education was not consistent across age groups. Out of 384,855 adults with smoking history in the cohort, 90,580 (23.5%) were current smokers and 23,787 (6.2%) were ex-smokers. Current smokers reported significantly less TB history, with 23% (OR: 0.77) less than those who had never smoked.

**Table 1:** Comparison of the characteristics of the total cohort, 486, 341 subjects, with those having a past history for TB, 5,036 subjects

	Total cohort	Self reported TB history (+)	(%)‡	OR†	(95%C.I.)
**Age**	486,341	5,036	(1.20)		
20-29	113,361	455	(0.45)	1	-
30-39	149,192	1,072	(0.81)	1.74*	(1.6~1.9)
40-49	87,217	981	(1.30)	2.77*	(2.5~3.1)
50-59	71,350	958	(1.60)	3.44*	(3.1~3.9)
60-69	47,610	967	(2.54)	5.05*	(4.5~5.6)
70+	17,611	603	(4.38)	8.39*	(7.4~9.5)
**Gender**	486,341	5,036	(1.20)		
Women	255079	1918	(0.88)	1	-
Men	231262	3118	(1.55)	1.79*	(1.7~1.9)
**Education**	399,135	4,969	(1.24)		
High school or above	291,711	3,364	(1.15)	1	-
Middle school or below	107,424	1,605	(1.49)	0.48*	(0.4~0.5)
**Smoking**	384,855	4,768	(1.24)		
Non smokers	270,488	3,148	(1.16)	1	-
Ex smokers	23,787	561	(2.36)	1.17*	(1.1~1.3)
Current smokers	90,580	1,059	(1.17)	0.77*	(0.7~0.8)
**Drinking**	379,996	4,720	(1.24)		
Nondrinker	296,235	3,349	(1.13)	1	-
Ex-drinker	11,599	249	(2.15)	1.16*	(1.0~1.3)
Occasional drinker	56,050	848	(1.51)	1.04	(1.0~1.1)
Regular drinker	16,112	274	(1.70)	1.15*	(1.0~1.3)
**Body Mass Index**	485,080	5,031	(1.20)		
<18.5 kg/m^2^	39,565	575	(1.62)	1.78*	(1.6~2.0)
18.5 -< 23 kg/m^2^	214,250	2,452	(1.32)	1	-
23 -< 25 kg/m^2^	98,734	1,007	(1.19)	0.65*	(0.6~0.7)
25 -< 30 kg/m^2^	113,897	904	(0.94)	0.47*	(0.4~0.5)
≧30 kg/m^2^	18,634	93	(0.59)	0.34*	(0.3~0.4)
**Diabetes**	483,641	5,036	(1.20)		
No history of diabetes	476,434	4,827	(1.18)	1	-
Known diabetes or on medications	9,907	209	(2.11)	1.17*	(1.0~1.4)

*: p < 0.05

† : OR: the likelihood of having TB history for those with a given variable against those without the given variable, adjusted for age

‡ The actual number of available subjects was used to arrive at the percentages. Questionnaires were administered as of 1996. Thus, during the start-up period of two years, 1994-1996, data on a proportion of subjects were missing. TB history (14%), education (18%), smoking (21%), and drinking (21%).

BMI and diabetes were more than 99% complete for total cohort.

Self-reported TB history increased when drinking increased. A lower BMI was associated with higher reported TB history. Those with known diabetes reported more TB history by 17%, compared against those with no diabetes.

### Smoking status and TB mortality risks

Table 2 shows the hazard ratios by smoking status on total cohort, and on sub-cohort with or without self-reported TB history for TB mortality. Compared with those who had never smoked, current smokers showed significantly increased deaths from TB, 3.37 (95% CI: 1.4~8.1) for current smokers (when age, gender, education and drinking, BMI and diabetes history were adjusted). The group that reported smoking the least, those smoking less than half a pack a day (HR = 3.70), those smoking less than 10 years (HR = 2.38), or those smoking less than 15 packs per year (HR = 2.54) showed substantially increased TB mortality risks.

**Table 2:** Hazard ratios (HR) by smoking status on total cohort, and on sub-cohort with self-reported TB history for TB mortality

	Total cohort (486,341)					Sub-cohort who responded to TB history question								
						**self reported TB history (-)** **414,460**		**Self reported TB history (+)** **5,036**			**Self reported** **TB history (-)** **414,460**		**Self reported** **TB history (+)** **5,036**	
					
	**N**	**HR†**	**(95%C.I.)**	**HR‡**	**(95%C.I.)**	**N**	**HR§**	**N**	**HR§**	**(95%C.I.)**	**HR||**	**(95%C.I.)**	**HR||**	**(95%C.I.)**

Hazard ratio- TB deaths	TB deaths (77)					64	**1**	13	5.90*	(3.1~11.2)				
Never smoker**	8	**1**	-	**1**	-	3	-	5	4.75*	(1.9~12.0)	**1**	-	29.47*	(6.9~125.2)
Current smoker**	21	4.19*	(1.8~9.7)	3.37*	(1.4~8.1)	17	-	4	10.10*	(3.3~30.8)	8.56*	(2.5~29.8)	44.02*	(9.6~201.6)
Ex-smoker**	6	1.90	(0.6~5.6)	2.02	(0.7~6.0)	3	-	3	5.51*	(1.6~18.4)	3.03	(0.6~15.3)	29.25*	(5.7~149.0)
Ever smoker**	27	3.97*	(1.9~8.4)	2.62*	(1.2~5.9)									
Current smoker**														
<half a pack/day	10	5.35*	(2.0~14.1)	3.70*	(1.4~10.0)									
≧half a pack/day	11	2.88*	(1.1~7.5)	1.84	(0.7~5.0)									
<10 years of smoking	1	3.01	(0.4~25.0)	2.38	(0.3~20.1)									
≧10 years of smoking	20	3.74*	(1.6~8.9)	2.44	(1.0~6.0)									
<15 pack-year	4	3.51*	(1.0~12.1)	2.54	(0.7~8.9)									
≧15 pack-year	17	3.83*	(1.6~9.3)	2.46	(1.0~6.2)									

*: p < 0.05 † : HR adjusted for age and gender, with nonsmokers as reference group;

‡ : HR adjusted for age, gender, education, drinking, BMI and DM history with nonsmokers as reference group

§ : HR adjusted for age, gender, education, drinking, BMI and DM history, with those with TB history (-) as reference group;

||: HR: adjusted for age, gender, education, drinking, BMI and DM history, with those nonsmokers with TB history (-) as reference group

** The actual number of subjects with smoking history was used to calculate HRs

For those with no prior TB history, the risk of dying from TB among current smokers increased more than nine-fold, with HR at 8.56 (95% CI: 2.5~29.8), when compared with those who had never smoked. When these smokers quit smoking, the ex-smokers' TB risk (HR = 3.03) reduced significantly by 65% compared to current smokers. [The hazard ratio between ex-smokers and current smokers in this case was 0.26 (95% CI: 0.1~0.9). Data not shown]. The TB risk of ex-smokers was not much different from those who had never smoked, both in the total cohort (HR = 2.02, 95% CI: 0.7~6.0) and in those with no TB history (HR = 3.03, 95% CI: 0.6~15.3).

The combined effect of smoking and TB history (HR = 44.02) in increasing TB mortality was larger than either smoking (HR = 8.56) or TB history alone (HR = 29.47), indicative of interaction between smoking and TB. When those with positive TB history were compared with those without, TB mortality increased by nearly 6-fold (5.90, 95% C.I.; 3.1~11.2). The risk of current smokers (HR = 10.10) doubled that of those who had never smoked (HR = 4.75) but reduced by 45% when they became ex-smokers (HR = 5.51) as the result of smoking cessation.

### Selected risk factors and TB mortality risks

Table 3 lists hazard ratios for selected risk factors related to TB mortality other than smoking. From the national data, the age-specific TB mortality rate increased sharply with increasing age, by approximately 150-300 times between age 20-29 and age 70+. Men had much higher rates than women, and, for age 60-69, the difference was approximately five-fold. Lower educated patients (middle school or below) had a TB mortality rate approximately three times more than higher educated patients. Drinking, significantly related to TB history, was also related to TB mortality, but did not reach the significance level. Low BMI patients, <18.5 kg/m^2^, had nearly a 10-fold increase in mortality, but overweight or obese patients were associated with reduced mortality. Diabetic patients, combining known cases and those discovered through screening, had 89% increased mortality from TB. The smoking attributable fraction for TB, when projected to Taiwan as a nation, was 37.7% (Table 4), given the national prevalence of 25.6% for current smokers [25].

**Table 3:** Hazard ratios (HR) for selected risk factors related to TB mortality other than smoking

	N	Number of TB deaths	TB mortality in Taiwan (1994 -- 2007) §		HR†	(95%C.I.)	HR‡	(95%C.I.)
**Age**	486,341		Male	Female				
20-29	113,361	1	0.3	0.2				
30-39	149,192	2	1.4	0.5				
40-49	87,217	2	3.3	0.8				
50-59	71,350	11	7.7	1.7				
60-69	47,610	24	25.9	5.3				
70+	17,611	37	102.6	32.9				
**Gender**	486,341							
Women	255079	12			1	-	1	-
Men	231262	65			5.69 *	(3.1~10.5)	8.39 *	(1.7~40.3)
**Education**	399,135							
High school or above	291,711	6			1.00	-	1.00	-
Middle school or below	107,424	30			3.38 *	(1.4~8.2)	2.51 *	(1.0~6.3)
**Drinking**	379,996							
Nondrinker	296,235	16			1	-	1	-
Ex-drinker	11,599	4			1.73	(0.6~5.3)	1.49	(0.5~4.6)
Occasional drinker	56,050	12			1.74	(0.8~3.8)	1.41	(0.6~3.2)
Regular drinker	16,112	2			1.24	(0.3~5.5)	0.90	(0.2~4.1)
**Body Mass Index**	485,080	77						
<18.5 kg/m^2^	39,565	22			4.98 *	(2.9~8.6)	9.67 *	(4.3~21.7)
18.5 -< 23 kg/m^2^	214,250	33			1	-	1	-
23 -< 25 kg/m^2^	98,734	7			0.29 *	(0.1~0.7)	0.39	(0.1~1.4)
25 -< 30 kg/m^2^	113,897	12			0.40 *	(0.2~0.8)	0.43	(0.1~1.4)
≧30 kg/m^2^	18,634	3			0.96	(0.3~3.1)	1.17	(0.1~9.1)
**Diabetes**	486,341							
No diabetes (by history or by screening)	462,351	63			1	-	1	-
Screened|| or known diabetes or on medications	23,990	14			1.76	(1.0~3.2)	1.89 *	(1.1~3.4)

*: p < 0.05

† : HR adjusted for age and gender

‡ : HR: adjusted for age, gender, education, drinking and smoking

§: Age-specific mortality rate in Taiwan (annual per 100,000) was shown here instead of directly calculating mortality from the cohort because the number of TB deaths in this cohort followed up even for 14 years was too small to be stable.

||: Fasting glucose ≧ 126 mg/dL

**Table 4:** Smoking attributable fraction for tuberculosis mortality

	N	Smoking Prevalence‡	HR†	95% CI	Smoking Attributable Fraction §
Current smoker	90,580	25.55%	3.37	(1.4~8.1)	37.7%
Ex-smoker	23,787	3.65%	2.02	(0.7~6.0)	6.9%
Non- smoker	270,488		1.00		

Total					**44.6%**

† : HR adjusted for age, gender, education and drinking, BMI and DM history with nonsmokers as reference group (from table 2)

‡ : Smoking prevalence from Wen et al. [25]

§: Smoking attributable fraction = Prevalence*(HR - 1)/{1 + [Prevalence*(HR - 1)]}

## Discussion

Smokers had very high TB mortality, as much as nine times those who had never smoked, but once they quit, the risk reduced substantially and was similar with those who never smoked. The 65% reduction of TB mortality risk as the result of smoking cessation was substantial and statistically significant. That TB risk could be reduced by nearly two-third if one quits smoking is a compelling evidence in highlighting the important role of smoking in TB control [26]. As smoking was responsible for more than one-third of TB deaths in Taiwan (37.7%), successful tobacco control in reducing smoking could favorably impact the TB mortality rate and reduce nearly one-third (30.7%) of this public health burden, which has long plagued the Taiwanese population. This large public health impact will be even larger, when applied to countries like China, where the prevalence of smoking and rates for TB are higher. Smoking cessation has been demonstrated for reducing TB incidence [27], but this study extended the benefit of smoking cessation on reducing TB mortality. With two-thirds of Chinese males smoking [21] and about three million TB cases, our observation should change the course of practice guidelines for managing active TB in the Chinese population. This is re-confirmed by our finding that smoking substantially aggravated TB mortality risks among those with TB history (from HR = 4.75 to HR = 10.10).

The size of the hazard ratio, 3.37 among the entire cohort or 8.56 among those with no TB history, is larger than those relative risks reported outside of India, ranging from 1.03-2.54 [2]. The magnitude of smoking risks and the substantial reduction upon quitting found from this cohort study has important practical implications. First, the lukewarm attitude by the clinicians toward the TB/smoking relationship will substantially improve, because the evidence is now based on mortality and not just active TB disease. The finding in this cohort that smoking worsened the rate of TB mortality and cessation reduced TB mortality will send a different message and may change clinicians' perspectives toward smoking cessation. Second, previous studies established a relationship between smoking and TB mostly based on case control studies where the selection of appropriate controls, a critical element in the study process, has been difficult. A review article found this problem ubiquitous in TB/smoking studies and emphasized that (hospital- or clinic-based sampling may lead to over-representation of smokers among the controls, thereby biasing the results toward the null) [2]. This analysis explains, at least in part, the size of the risks identified in this study were larger than those from case control [1]. Third, our data distinguishing those who reported a history of TB from those unaware, showing similar but graded mortality risks, and the substantial benefits observed for both groups added strength and provided consistency to the TB/smoking relationship.

That more than one-third of TB deaths were attributable to smoking (37.7%) adds to the long litany of smoking hazards in Asian countries [24]. The two calculations of TB deaths attributable to smoking reported from India, 61% [18], or 38% (males) [28], were both based on a case control study, and 32.8%, reported from Hong Kong [11], on incidence and not mortality. By and large, our calculation of the deaths attributable to smoking, 37.7% for current smokers, was not that much different from those reported for active diseases.

One surprising finding was that significantly fewer smokers reported TB history and proportionately more unaware of having TB than those who had never smoked. On the surface, this appeared to contradict the notion that smokers have more TB infection [17]. This paradoxical phenomenon, however, confirmed our fear that smokers were less vigilant regarding the potential onset of TB. Partly because of fewer contacts with medical professionals, and partly because TB and smoking shared many clinical symptoms, smokers were less informed of the TB risk. This oversight, by smokers and their doctors alike, has important implications, because TB in smokers in this society was found at a later stage or in a more serious condition than in those who had never smoked, leading to the higher mortality rate [1].

In addition to smoking, passive smoking is a potential confounder [29] and in the absence of these data, their impact on susceptibility to TB infection is unknown. However, the concentrations of second-hand smoke to which humans are exposed to are considered to be of far less than that of active smoking. Alcohol drinking has also been reported to be an independent risk factor for active TB [30,31]. We observed that drinking was associated with more TB history and a tendency to increase subsequent TB mortality in all categories of drinkers, including occasional, ex- and regular drinkers. The failure to reach significance in mortality can be the result of inadequate sample size, but the predominant style of social drinking with meals in Taiwan may have reduced the drinking risks as previously reported from India [31].

The implication that smoking increased TB mortality and quitting reduced that risk can be explained in part by the known hazards of smoking and in part by the delayed treatment for smokers with TB. Smokers' medical conditions often can be complicated by the presence of co-morbidities, such as chronic obstructive pulmonary disease (COPD), increased infection with reduced immunity, or malnutrition. COPD may play an important role in the relationship between smoking and TB. As a large number of smokers develop COPD, which impairs the respiratory system and masks the physical signs of TB, the smokers may become less aware of TB. At the same time, in patients with both TB and smoking-related COPD, the severity of disease may be aggravated, which may accelerate mortality and lead to increased TB mortality among smokers as shown in this study. Furthermore, the underweight individuals (BMI < 18.5 kg/m^2^) observed in our study were associated with a 10-fold increase in TB mortality. Similar observations have been previously reported in Hong Kong and India [32,33]. Elimination of smoking would improve the lung function, reduce infection, restore immunity and improve general well-being. Another implication is delayed treatment of smokers with TB in the clinical setting [34] as smoking and TB share similar clinical symptoms and clinicians were misled by smoking symptoms. When smokers quit smoking and become ex-smokers, there will be less confusion of symptoms.

This study was the first to associate diabetes with increased TB mortality (HR = 1.89), although the relationship with active TB was well known[35]. A review of 13 observation studies found the positive relationship between diabetes and the risk of getting active TB[35], with HR between 3.11 in one group and 1.62 in another, with the latter nearly identical with the one in this study. It is of concern to note that a large portion of the diabetes cases (59%) in this study were unaware of their diabetes, and therefore, less aware of their TB risks (Table 1).

There are important limitations in this study. First, the cohort came from a higher social class with the ability to pay for the screening. Therefore, it may not be fully representative of the society. For national prevalence of smoking or TB, cohort data needs to be standardized by the socioeconomic status as in our previous reports. However, as prevalence was not the focus of this study, and hazard ratios or odds ratios were internally standardized, the observed increase in smokers' TB risks can be a reasonable estimate of the general population. In the same vein, we used national smoking rates in calculating the smoking attributable TB mortality, and thus, the resulting estimate should also be valid. Second, mortality from TB was determined based on death certificates and not confirmed in any other way. The likely direction of error should come more from omission than commission, i.e. from deaths assigned to causes other than TB when TB was the underlying cause. For example, if an HIV positive patient died with TB, HIV is more likely to be recorded as the cause of death [36]. Consequently, the hazard ratios and the percentage of deaths attributable to smoking, 41.4%, developed in this study could have been under-estimated. Third, a number of TB deaths did not record smoking status in the early part of the cohort recruitment. This has substantially reduced the number of TB deaths associated with smoking. However, our conclusion was based on the actual data, significance was found after statistical treatment, even though limited sample size failed to show dose response relationship between smoking quantity or intensity and TB mortality risks in Table 2. Fourth, TB history data were self-reported and not clinically confirmed. While under-reporting of TB history could have existed, most of them should be unintentional, as participants in this study, having paid a sizable fee to be screened, were genuinely interested in uncovering medical problems and thus would volunteer as much medical information as possible for an accurate evaluation. As there is no reason for smokers to have different reporting mechanism from never smokers, our study result showing a low awareness of TB among smokers in this cohort were valid. The value of this TB history became obvious when a striking difference in mortality outcome was found. Fifth, the benefits of smoking cessation were assumed in this study by comparing the difference in TB mortality between smokers and ex-smokers as separate groups and not in a before/after manner for the given smokers. However, this assumption is commonly made in tobacco research and this comparison method is widely practiced. In general, smokers don't change their behavior patterns unless there is a pressing medical condition. Some participants may be smokers at the enrollment and later on quit smoking, and vice versa. However, the proportions of people started or quit smoking are relatively low. Moreover, some low-risk participants were misclassified as high-risk participants and thus the relative risk for TB mortality calculated in this study would more likely be over-estimated.

To assess the benefits in a before/after comparison requires clinical trials on smokers randomly assigned to cessation/no cessation group. Because of the paucity of TB deaths and the associated ethical considerations, conducting such a trial would be a monumental challenge.

## Conclusion

Smokers had a very high TB mortality rate, as much as nine times higher than those who had never smoked, but once they quit, the TB mortality rate reduced substantially (65%) and was similar to those who never smoked. Smoking cessation has benefits to the smoking individual far beyond reducing TB risk, but successful tobacco control could favorably impact the TB mortality rate and reduce this public health burden, as smoking cessation could reduce nearly one-third of tuberculosis deaths. Clinicians should be more alert about reducing the TB risk among smokers, since smokers and TB patients share clinical symptoms and TB among smokers was more difficult to identify than among never smokers.

## Competing interests

The authors declare that they have no competing interests.

## Authors' contributions

CPW designed the study, conceived the idea and drafted the article. TCC, HTC, MKT analyzed and interpreted the data. TYC and SPT provided statistical advice and critically revised the article for important intellectual content. CPW had final approval of the article. All authors read and approved the final manuscript.

## Pre-publication history

The pre-publication history for this paper can be accessed here:

http://www.biomedcentral.com/1471-2334/10/156/prepub
